# Analgesia in the Emergency Department for Lower Leg and Knee Injuries: A Case Report

**DOI:** 10.5811/cpcem.7201

**Published:** 2025-01-16

**Authors:** Michael Shalaby, Yonhoon Lee, Joseph McShannic, Michael Rosselli

**Affiliations:** * Herbert Wertheim College of Medicine at Florida International University, Department of Emergency Medicine, Miami Beach, Florida; † Mount Sinai Medical Center Miami Beach, Department of Emergency Medicine, Miami Beach, Florida

**Keywords:** saphenous, adductor canal, popliteal sciatic, regional anesthesia, lower limb, fracture

## Abstract

**Introduction:**

Lower extremity injuries are commonly evaluated and treated in the emergency department (ED). Pain management for these injuries often consists of acetaminophen, non-steroidal anti-inflammatories, and opioids. Despite this treatment regimen, adequate analgesia is not always achieved.

**Case Report:**

A 38-year-old man presented to the ED with a non-displaced tibia-fibula fracture. The patient did not attain analgesia with intravenous medications but did get complete anesthesia of his lower leg with a combination saphenous and popliteal sciatic nerve block.

**Conclusion:**

Emergency physicians possess the skill set required to effectively perform a saphenous and popliteal sciatic nerve block and should consider adding this procedure to their armamentarium of pain management techniques in treating injuries distal to the knee.

## INTRODUCTION

Lower extremity (LE) injuries account for nearly 15% of emergency department (ED) visits yearly, with trauma to the knee and distal comprising an overwhelming majority (greater than 75%).^1^ Lower extremity injuries are painful, particularly fracture-dislocations.^2^ Analgesia for LE injuries is highly variable in time to administration, dosing, and adequacy. For example, patients with LE injuries tend to wait longer than average for analgesics (especially ambulatory patients).^3^ Moreover, even when treated with opioids, most patients with serious LE injuries do not attain adequate pain control in the ED.^4^ Opioids also lead to complications such as nausea, vomiting, hypotension, and respiratory depression.^5^ Elderly patients with LE injuries are especially susceptible to increased mortality and morbidity,^6^ perhaps partly due to the administration of opioids.

Lower extremity limb injuries requiring inpatient hospitalization can lead to significant financial, psychosocial, and quality-of-life burdens for patients, which extend far beyond the hospital stay.^7^ Herein we present the case of a patient with a combined tibia-fibula fracture with intractable pain despite significant amounts of opiate analgesics, but who achieved complete anesthesia with saphenous and popliteal sciatic nerve blocks.

## CASE REPORT

A 38-year-old male presented via emergency medical services (EMS) after sustaining a right lower leg injury from falling off a skateboard. The lower leg had no visible deformity, but the patient was in severe pain, which he described as the worst of his life. He had received 10 milligrams (mg) of intramuscular morphine by EMS without improvement. Given his significant pain level, upon arrival to the ED he was given 1 mg of intravenous (IV) hydromorphone, which was repeated 15 minutes later with minimal improvement. The patient subsequently received two separate doses of 0.1 mg per kilogram of IV ketamine, after which his pain was minimally relieved. A radiograph was performed and showed a tibia-fibula fracture. The patient had soft LE compartments, full sensation, and 2+ dorsalis pedis and posterior tibial pulses, so there was no concern for acute compartment syndrome.

After minimal relief with opioids and ketamine, the patient consented to an adductor canal and a popliteal sciatic block. The adductor canal block was performed with 15 milliliters (mL) bupivacaine 0.5% without epinephrine, and the popliteal sciatic block was performed with 10 mL bupivacaine 0.5% without epinephrine. Within 10 minutes, the patient noted complete resolution of his pain and ironically opted to leave against medical advice instead of being admitted for future pain control and operative planning. On follow-up with the patient one week later, he noted that the anesthetic lasted about 14 hours and that he had presented to another hospital two days later where he underwent successful and uncomplicated open reduction and internal fixation of his injury.

## DISCUSSION

### Anatomy of the Saphenous Nerve

The saphenous nerve is the largest cutaneous branch of the femoral nerve,^8^ consisting of purely sensory neurons without a motor component.^9^ The saphenous nerve provides sensation to the patella, the medial femoral and tibial condyles, and the medial malleolus (Figure). The saphenous nerve courses immediately lateral to the femoral artery in the distal thigh between the adductor longus and vastus medialis muscles, a potential space known as the “adductor canal.” Thus, the saphenous nerve block is synonymous with the “adductor canal block.” Although the saphenous nerve is difficult to visualize directly on point-of-care ultrasound (POCUS), it can be presumed to course immediately anterolateral to the femoral artery in the middle to medial lower third of the thigh. This view is already familiar to most emergency physicians who perform POCUS for deep vein thrombosis of the LE. Most commonly, the adductor canal can be visualized anywhere from the middle anterior to the lower medial third of the thigh based on patient anatomy.

**CPC-EM Capsule**

What do we already know about this clinical entity?

*Saphenous and sciatic nerve blocks have been well documented for use in emergency medicine*.

What makes this presentation of disease reportable?

*Used together as a form of dense anesthesia, these nerve blocks proved effective for rapid pain relief in a patient with a non-displaced tibia-fibula fracture*.

What is the major learning point?

*Saphenous and sciatic nerve blocks are relatively straightforward to perform and effective for pain control*.

How might this improve emergency medicine practice?

*Lower extremity injuries are painful. These nerve blocks can provide emergency physicians with the tools to alleviate pain from any injury distal to and including the knee*.

### Anatomy of the Sciatic Nerve

**Image 1:**
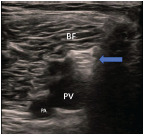
Transverse ultrasound view for popliteal sciatic nerve block. *BF*, biceps femoris muscle; *PV*, popliteal vein; *PA*, popliteal artery. Blue arrow: sciatic nerve.

The sciatic nerve has a unique architecture. It is comprised of the tibial nerve and the common peroneal nerve, each with its own epineurium, surrounded by a paraneural sheath.^10^ These two nerves diverge from each other in the popliteal fossa, where the popliteal sciatic nerve block is performed. The sciatic nerve provides sensory innervation to the rest of the lower leg not covered by the saphenous nerve, including the lateral calf and the entire foot (Figure). Unlike the saphenous nerve, the sciatic nerve also has a motor component, which imparts function to all the muscles of the lower leg and the foot. The popliteal, or “distal,” sciatic nerve can be visualized in the popliteal fossa, usually superficial to the popliteal vein (Image 1). The paraneural sheath, which surrounds the sciatic nerve, is visible as a hyperechoic fascial layer separating the nerve from the surrounding musculature. Physicians may also be familiar with POCUS of the sciatic nerve since it is the same view for the popliteal vein component of the deep vein thrombosis exam.

### Ultrasound-Guided Adductor Canal Block

**Image 2:**
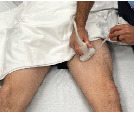
Patient positioning for an adductor canal (saphenous nerve) block. The needle’s trajectory is lateral to medial.

**Image 3:**
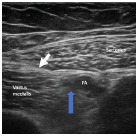
Ultrasound view for saphenous nerve block. *FA*, femoral artery. White arrow: adductor canal. Blue arrow: anatomic location of saphenous nerve.

To perform the saphenous nerve block, the patient should be supine (Image 2). The femoral artery should be visualized within the middle of the screen, with the adductor canal lateral to it (Image 3). From anterolateral to posteromedial, a spinal needle is advanced in-plane to the transducer. To ensure that no anesthetic is wasted, the physician should first hydrodissect the adductor canal with normal saline to visualize the “unzipping” of the fascial plane prior to instilling anesthetic. The procedure may be performed with a variety of anesthetics depending on treatment goals: bupivacaine 0.5% and ropivacaine 0.5% provide anesthesia on the order of hours to days, while anesthesia from lidocaine 1% usually lasts less than three hours.

### Ultrasound-Guided Popliteal Sciatic Nerve Block

**Image 4:**
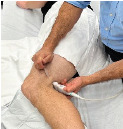
Patient positioning for a distal sciatic nerve block. The needle’s trajectory is lateral to medial.

For physicians performing a popliteal sciatic block, we recommend first blocking the saphenous if the patient is already supine, and then allowing the patient to turn to lateral decubitus with the affected leg up (Image 4). Patients do not have to be prone, which may be difficult with LE injuries. Given its depth in most patients, the popliteal sciatic nerve block should also be performed with a spinal needle in a lateral-to-medial trajectory. The sciatic nerve is usually visualized immediately superficial to or adjacent to the popliteal vein (Image 1). The most crucial aspect of the popliteal sciatic block is to instill anesthetic within the surrounding paraneural sheath, which provides denser and faster blockade.^10^, ^11^ As with the saphenous nerve block, bupivacaine and ropivacaine impart longer lasting anesthesia compared to lidocaine. While it is common for physicians to block immediately at the bifurcation of the common peroneal and tibial nerves, blocking proximally to the bifurcation has been successfully described.^12^ Blockade proximally may be technically easier and equally effective since it allows for a larger target than at the exact point of bifurcation. Physicians must provide crutches to any ambulatory patient receiving a popliteal sciatic block since the block will result in lower leg paralysis.

Emergency physicians regularly treat patients with LE limb injuries. Frequent opioid analgesic administration for such patients carries high complication rates and does not guarantee adequate analgesia. Lower extremity injuries impose significant costs in both hospital charges and days lost of production, as well as psychosocial burdens.^7^ The saphenous nerve block combined with the popliteal sciatic block is a powerful tool for physicians to treat and eliminate any pain from the knee down. Both blocks boast relatively straightforward sonoanatomy, with which physicians who are proficient with POCUS may already be familiar. Furthermore, while each block carries intrinsic risks such as nerve damage, vascular puncture, and local anesthetic systemic toxicity (as with all methods of regional anesthesia), these techniques are relatively safe given the lack of risky anatomic structures nearby, such as the lungs or carotid arteries with brachial plexus blocks.

In our experience, both the saphenous nerve block and the popliteal sciatic block are relatively quick procedures that can be performed within a few minutes each. Additionally, if long-acting anesthetics such as bupivacaine or ropivacaine are employed for blockade, patients can experience hours to days of anesthesia. Thus, regional anesthesia in general can reduce patients’ use of opioids. Moreover, time- and labor-intensive procedural sedation and anesthesia, which carries risks of respiratory depression, hypotension, and vomiting, can be avoided for LE fractures requiring reduction.^13^ Lastly, the use of regional anesthesia for patients with LE injuries and exam findings concerning for compartment syndrome (such as significant edema, tenderness, altered sensation, coolness to touch, or pulselessness) is controversial. While the American Society of Regional Anesthesia does not oppose the use of regional anesthesia in suspected compartment syndrome, citing that compartment pressure measurement is the most accurate method for determining the need for emergent fasciotomy,^14^ emergency physicians should consult with their surgical team before performing regional anesthesia, as this may disguise worsening compartment syndrome and the need for emergent fasciotomy.

## CONCLUSION

Lower extremity limb injuries are common and can be quite painful. The combined saphenous nerve block and popliteal sciatic blocks can provide dense anesthesia to the lower extremity from the knee down. Emergency physicians who are familiar with in-plane needle-guided procedures (such as ultrasound-guided peripheral IV lines) possess the skill set required to effectively perform a saphenous and popliteal sciatic nerve block and should consider adding this procedure to their multimodal approach to analgesia for injuries distal to the knee.

## Figures and Tables

**Figure:**
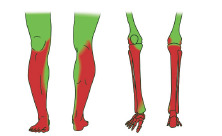
Tissue and osseous sensory distributions of saphenous and popliteal sciatic nerves. Red color: sensory distribution of popliteal sciatic nerve. Green color: sensory distribution of saphenous nerve. Image courtesy of Anthony Casazza.

## References

[1] Lambers K, Ootes D, Ring D (2012). Incidence of patients with lower extremity injuries presenting to US emergency departments by anatomic region, disease category, and age. Clin Orthop Relat Res.

[2] Clapp ADM, Thull-Freedman J, Mitra T (2020). Patient-reported pain outcomes for children attending an emergency department with limb injury. Pediatr Emerg Care.

[3] Abbuhl FB, Reed DB (2003). Time to analgesia for patients with painful extremity injuries transported to the emergency department by ambulance. Prehosp Emerg Care.

[4] Neighbor ML, Honner S, Kohn MA (2004). Factors affecting emergency department opioid administration to severely injured patients. Acad Emerg Med.

[5] Ramadan M, Alnashri Y, Ilyas A (2022). Assessment of opioid administration patterns following lower extremity fracture among opioid-naïve inpatients: retrospective multicenter cohort study. Ann Saudi Med.

[6] Sharfman ZT, Parsikia A, Rocker TN (2021). Increased morbidity and mortality in elderly patients with lower extremity trauma and associated injuries: s review of 420,066 patients from the National Trauma Database. Injury.

[7] Dischinger PC, Read KM, Kufera JA (2004). Consequences and costs of lower extremity injuries. Annu Proc Assoc Adv Automot Med.

[8] Sebastian MP, Bykar H, Sell A (2019). Saphenous nerve and IPACK block. Reg Anesth Pain Med.

[9] Rasouli MR, Viscusi ER (2017). Adductor canal block for knee surgeries: an emerging analgesic technique. Arch Bone Jt Surg.

[10] Karmakar MK, Reina MA, Sivakumar RK (2021). Ultrasound-guided subparaneural popliteal sciatic nerve block: there is more to it than meets the eyes. Reg Anesth Pain Med.

[11] Perlas A, Wong P, Abdallah F (2013). Ultrasound-guided popliteal block through a common paraneural sheath versus conventional injection: a prospective, randomized, double-blind study. Reg Anesth Pain Med.

[12] Tran DQH, González AP, Bernucci F (2013). A randomized comparison between bifurcation and prebifurcation subparaneural popliteal sciatic nerve blocks. Anesth Analg.

[13] Shalaby M, Smith M, Tran L (2023). Utility of supraclavicular brachial plexus block for anterior shoulder dislocation: could it be useful?. West J Emerg Med.

[14] Lam D, Pierson D, Salaria O (2023). Pain control with regional anesthesia in patients at risk of acute compartment syndrome: review of the literature and editorial view. J Pain Res.

